# Regmex: a statistical tool for exploring motifs in ranked sequence lists from genomics experiments

**DOI:** 10.1186/s13015-018-0135-2

**Published:** 2018-12-08

**Authors:** Morten Muhlig Nielsen, Paula Tataru, Tobias Madsen, Asger Hobolth, Jakob Skou Pedersen

**Affiliations:** 1 Department of Molecular Medicine (MOMA), Aarhus University Hospital, Palle Juul-Jensens Boulevard 99, 8000 Aarhus C, Denmark; 2 Bioinformatics Research Centre, Aarhus University, C.F. Møllers Allé 8, 8000 Aarhus C, Denmark

## Abstract

**Background:**

Motif analysis methods have long been central for studying biological function of nucleotide sequences. Functional genomics experiments extend their potential. They typically generate sequence lists ranked by an experimentally acquired functional property such as gene expression or protein binding affinity. Current motif discovery tools suffer from limitations in searching large motif spaces, and thus more complex motifs may not be included. There is thus a need for motif analysis methods that are tailored for analyzing specific complex motifs motivated by biological questions and hypotheses rather than acting as a screen based motif finding tool.

**Methods:**

We present Regmex (REGular expression Motif EXplorer), which offers several methods to identify overrepresented motifs in ranked lists of sequences. Regmex uses regular expressions to define motifs or families of motifs and embedded Markov models to calculate exact p-values for motif observations in sequences. Biases in motif distributions across ranked sequence lists are evaluated using random walks, Brownian bridges, or modified rank based statistics. A modular setup and fast analytic *p* value evaluations make Regmex applicable to diverse and potentially large-scale motif analysis problems.

**Results:**

We demonstrate use cases of combined motifs on simulated data and on expression data from micro RNA transfection experiments. We confirm previously obtained results and demonstrate the usability of Regmex to test a specific hypothesis about the relative location of microRNA seed sites and U-rich motifs. We further compare the tool with an existing motif discovery tool and show increased sensitivity.

**Conclusions:**

Regmex is a useful and flexible tool to analyze motif hypotheses that relates to large data sets in functional genomics. The method is available as an R package (https://github.com/muhligs/regmex).

**Electronic supplementary material:**

The online version of this article (10.1186/s13015-018-0135-2) contains supplementary material, which is available to authorized users.

## Introduction

Motif discovery is a classical problem in sequence analysis and its scope broadens with modern sequencing technologies. A large number of tools are designed to find enriched motifs in sequences, with the majority aimed at finding motifs that are enriched in a foreground set of sequences relative to a background set. This is optimal for sequences where a binary variable defines a foreground and a background. However, many experimental settings are associated with continuous variables where set-based methods are suboptimal. Instead of using a hard threshold to divide a continuous variable into foreground and background, it is more powerful to take the magnitude of the continuous variable directly into account.

In the past two decades, motif enrichment methods have been developed that can exploit the ranking in a list of sequences, e.g. [1–9]. These methods seek to find the motifs that best correlate with the rank. Most commonly, this is achieved by exhaustively searching through the space of all simple motifs of a given length k (k-mers). K-mers, ranked by their correlation measures, are then output directly; clustered and used to define position weight matrices (PWMs); or used as seeds in a variety of downstream algorithms to refine the top correlating motifs.

A general challenge of motif analysis, and specifically of methods based on an exhaustive search, is the rapid increase in search space with motif size and complexity. This problem has been addressed by using suffix trees, allowing exhaustive searches of large spaces such as all variable gap motifs up to a given length [4]. However, a functional motif may display a high degree of complexity that current methods does not meet. For example, many snoRNAs are known to bind their targets at two sites separated by a variable number of nucleotides leading to a composite motif. In addition, regulation of biological systems often relies on multiple factors acting in concert. For instance, endogenous RNAs have been shown to perturb regulatory networks consisting of multiple miRNAs [10]. It is thus valuable to be able to evaluate enrichments for a motif defined as subsets of binding sites in combination, as well as arbitrarily complex motifs, in a hypothesis driven way. Regular expressions are well suited to specify composite motifs even with variable gaps. Because of the large search space, regular expressions are not attractive for motif discovery algorithms, but for testing concrete hypotheses they are well justified. There are currently no tools available to calculate enrichment of motifs defined as regular expressions in ranked sequences, and we thus developed Regmex for this purpose.

A central aspect in motif analysis of ranked sequences is the significance evaluation of the motif rank correlation. A number of approaches have been used, including linear regression models [11], Wilcoxon rank sum tests [12], a Kolmogorov–Smirnov based approach [8], a Brownian bridge based approach [2], and methods that use variants of hyper geometric tests [1, 4, 9]. The various methods also have different approaches for motif scoring. Examples include simple presence/absence scores for each sequence [8, 9]; dependence of sequence lengths and global base composition [1]; and probabilistic scoring that models base composition of every sequence in the rank list [2]. Presence/absence scores in particular suffer a risk of bias because sequence length and composition is not included in the score model, which is a problem if e.g. sequence lengths are biased in the rank. Also, presence/absence score-based methods may be underpowered in situations where the number of motif occurrences in a single sequence matters.

**Fig. 1:**
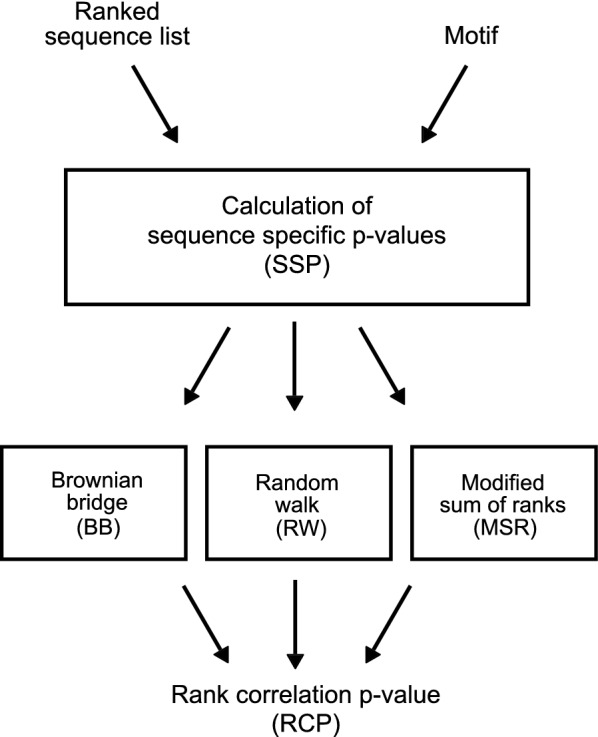
Flow diagram of the procedures for calculating sequence specific p-values and rank correlation or clustering p-value in Regmex

Based on these issues, we see a need for a tool that calculates accurate sequence dependent p-values for motif observations and that allows hypotheses for flexible motifs and motif combinations to be evaluated. We present Regmex, a motif enrichment tool, with a number of new features aimed at accurate significance evaluation. The tool is implemented in R and provide a simple interface to evaluate both concrete hypotheses about given motifs in small scale experiments, e.g. the ranking of sequences with miRNA target sites in a miRNA perturbation experiment, and performing computationally efficient screens of large and complex motif sets across many samples. Regmex makes use of two sequential steps (Fig. 1). First, sequence specific motif p-values are calculated, that depend on both sequence lengths and base compositions using an embedded Markov model. Similar ideas have been considered previously, yet not implemented in the context of ranked sequences [13–16]. Second, depending on the problem and hypothesis, motif correlation with rank or the tendency of motifs to cluster along the list of sequences can be evaluated in one of three different ways:

1. A Brownian bridge based approach (BB).
2. A modified sum of ranks method which takes sequence properties into account (MSR).
3. A random walk based method which is sensitive to clustering of motif observations anywhere in the sequence list (RW).

Regmex makes use of regular expressions for defining motif models. It thereby allows far more complex motifs than simple k-mers, e.g., consecutive arbitrarily spaced sub-motifs.

We illustrate some of Regmex’s possibilities using both simulated and real data sets, where we confirm previously reported results on miRNA target motifs in 3′UTR sequences of down-regulated genes in miRNA pertubation experiments. We further use Regmex’s capability to combine motifs and show that the presence of a U-rich motif (URM) strengthens this effect. This analysis further suggests that an upstream URM has more effect than a downstream one. Finally, we compare Regmex with two motif finding methods, Sylamer [1] and DRIMust [4], and find that Regmex and DRIMust have increased sensitivity over Sylamer while Sylamer is faster.

## Materials and methods

### Regmex

In this study, we introduce Regmex, a motif analysis tool available as an R package. Regmex is designed with flexibility in mind to study rank correlation or clustering of motifs in an ordered list of sequences.

Briefly, it takes as input a list of ranked DNA sequences, which could come from a genomics experiment, and one or more motifs, each defined as a regular expression (RE) (Fig. 1). The output, in its simplest form, contains the rank correlation or clustering p-values (RCPs) for the input motifs. Alternatively, it is possible to get the underlying sequence specific p-values (SSPs) for motifs as well as count statistics, etc.

To illustrate the power of REs in a biological sequence context, we consider the following examples:

1. A stem loop structure, TTTCNNNGAAA, found in the 3′UTR of many key inflammatory and immune genes [17]. Although this is a simple RE, it captures 64 11-mers in one expression, and Regmex reports the rank correlation p-value of the combined set.
2. A G-quadroplex structure, GGGLGGGLGGGLGGG, where L = (N|NN|NNN|NNNN).This is found e.g., in telomeric regions [18].
3. Any size open reading frame: ATG(NNN)*?((TGA)|(TAA)|(TAG)). This RE is an example of an enormous set, which would be difficult to obtain without an RE.

An advantage of REs is that they can capture any set of simple motifs. For example, a set of experimentally verified binding sequences can be expressed as a single RE, with matching and p-value evaluation based on exactly this set.

### Sequence specific motif p-value calculation

**Fig. 2:**
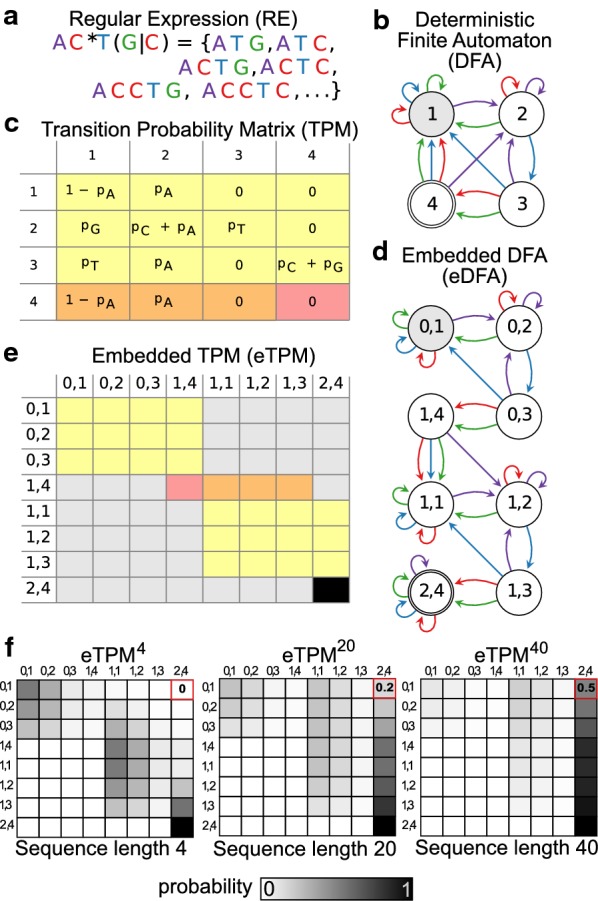
**a** Motif in the form of a regular expression. Base coloring applies throughout the figure. **b** Deterministic finite state automaton (DFA) corresponding to the regular expression in **a**. Initial state is indicated in gray, and end state is indicated by a double circle. **c** Transition state probability matrix (TPM) associated with the model in **b**. **d** Embedded Markov Model (eDFA) for two observed occurrences of the motif. States are pre-indexed with the number of already observed motifs. **e** Embedded transition state probability matrix (eTPM) associated with the eDFA. The yellow matrix is an exact copy of the yellow matrix from **c**. The gray entries have zero probability. The transition probabilities from the end state of the DFA model (red/orange entries in matrix from **c**) are shifted forward and contain the initial state of the next motif occurrence, except for any end to end transition probability (occurs for REs ending with a *), which remains in the DFA template (red entry). The final state of the eDFA ((2,4) in **d**) is an absorbing state with transition probability of 1 to itself, indicated in black. **f** Heat diagrams of the *n*-step eTPM reflecting the probability of moving between states in the eDFA, given a random sequence of length *n* with a specific base composition. The row corresponding to the initial state (0,1) holds the probability distribution of going from the start state to any state in the eDFA in *n* steps. The last entry of this row (red entry) holds the probability of the observed number of motifs (*n*_*obs*_) or more in the sequence (the SSP)

Regmex calculates a motif rank correlation p-value (RCP) based on sequence specific p-values for observing the motif the observed number of times (*n*_*obs*_) or more. Briefly, from a deterministic finite state automaton (DFA) associated with the regular expression motif, we derive a sequence specific transition probability matrix (TPM), which is used to build an embedded TPM (eTPM) specific for *n*_*obs*_ (Fig. 2). The SSP is subsequently read from the eTPM raised to the power of the sequence length. These steps are explained in more detail below.

### Deterministic finite state automaton

For any regular expression, the corresponding DFA can be built, which is the initial step in the SSP calculation (Fig. 2b). The DFA starts in an initial state, accepts symbols (i.e. nucleotides) on the edges and moves through the states. The end state corresponds to having observed the RE. The DFA used here recognizes an extended regular expression, as described in [19]. The routine used to build the DFA for a given regular expression is implemented in Java, using [20], and supports standard regular expression operations (concatenation, union and Kleene star) and overlaps.

### Markov embedding

The DFA graph structure can also be thought of as a Markov model, where instead of accepting symbols, it generates symbols on the edges with probabilities corresponding to the base frequencies in a given sequence. The Markov model can be represented by a transition probability matrix (TPM), which holds the probabilities of moving between states of the DFA upon observing bases from the sequence (Fig. 2c). The TPM raised to the power of n, TPM^n^, holds the probability of moving between states after observing n bases.

We are interested in the SSP and thus need to have a probability model that takes *n*_*obs*_ into account. Regmex does this by making a model expansion using the DFA as a template. We refer to this as an embedded DFA (eDFA) (Fig. 2d). Specifically, the template DFA is copied *n*_*obs*_ times and outgoing edges of the end state(s) of the DFA template are moved to the corresponding states in the next template copy. This effectively allows the embedded model to count how many times the RE motif has been observed. The final state of the eDFA is absorbing, so no further motif observations are scored.

Again, the eDFA can be thought of both as an automaton accepting symbols or as a Markov model generating symbols on edges. As above, Regmex constructs a transition probability matrix (eTPM) based on the eDFA (Fig. 2e). The eTPM^n^ holds probabilities of moving between states of the eDFA given a random sequence of length n with the observed base frequencies (Fig. 2f). We can now extract the probability distribution of the RE motif in a given sequence by reading the row corresponding to the initial state (0,1) in the eTPM^n^. In particular the probability of observing the motif *n*_*obs*_ number of times or more (the sequence specific p-value, SSP) can be read in the final state column of the initial state row (red field in Fig. 2f).

### Motif rank correlation p-value

**Fig. 3:**
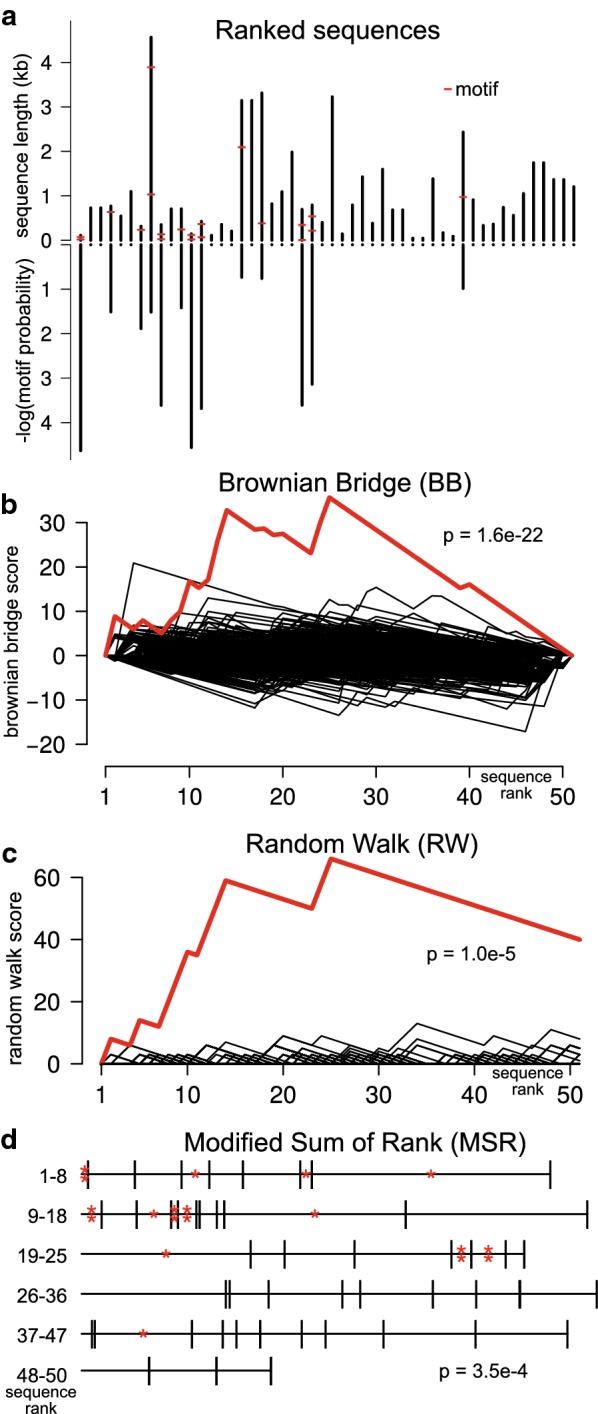
**a** Sequences enriched with a 7-mer motif (ACGTGAT) as indicated with red marks. Upper bars indicate sequence lengths, lower bars indicate SSPs for the motif. **b** Brownian bridge for the 7-mer motif in **a** (red) and for 500 random 7-mer motifs (gray). The RCP corresponding to the BB is indicated. **c** Random walk for the motif in **a** (red) and 500 random 7-mer motifs (black). The RCP corresponding to the RW is indicated. **d** Schematic of the MSR method. Lines represent sequences with lengths proportional to the probability of observing the motif one or more times. A motif occurrence is marked by an asterisk. The RCP corresponding to the motif distribution is indicated

In the downstream analysis, Regmex uses the calculated SSPs when calculating the RCP. In Regmex, we have implemented three methods for evaluating motif rank correlation or motif clustering, which have different strengths. These methods are based on Brownian bridge (BB), random walk (RW), and modified sum of rank (MSR) statistics. The concepts underlying these statistics are illustrated on a short list of 50 sequences with an enriched motif (Fig. 3). The bias in the distribution of motifs may vary depending on the analyzed problem and the choice of method used to evaluate the correlation may differ in detection power. E.g., one test may be well-powered for detecting long motifs occurring rarely in the sequence list and another for detecting frequent short motifs.

### Brownian bridge

This method is a re-implementation of the method developed by Jacobsen et al. [21] and recently implemented in cWords [2]. Our implementation differs in the calculation of the sequence specific p-values (SSPs) and in how we calculate the rank correlation p-value. The method calculates the max value *D* of a running sum of mean adjusted log scores of the SSPs

$$r_{i} = r_{i - 1} + ls_{i} - \overline{ls} ,$$

where $$ls_{i} = - ln\left( {p_{i} + \alpha } \right),$$$$\alpha$$ is a score dampening factor set to 10^−5^ and $$\overline{ls}$$ is the mean of the log scores.

The running sum starts and ends in zero and hence is a Brownian bridge under the null model (see Fig. 3b). We identify the rank correlation p-value from the analytical distribution of max values of a Brownian bridge [22].

$$Pr\left( {M \ge m} \right) = 1 - 2\sum\limits_{k = 1}^{\infty } {\left( { - 1} \right)^{k} e^{{ - 2k^{2} m^{2} /n}} },$$

where $n$ is the number of sequences in the sequence list.

### Random walk statistics

The random walk (RW) method is similar to the use of random walks in the BLAST algorithm [23]. This method is sensitive to clustering of motifs anywhere in the sequence list. The sequence specific p-values (SSPs) for a motif are transformed into steps in a walk (Fig. 3c). Under the null model the motif is not enriched and the SSPs follow the uniform distribution. The SSPs are transformed into steps according to a scoring scheme where small p-values (SSPs) correspond to a positive step and large p-values correspond to a negative step. The exact scoring scheme is based on assumed motif densities in the foreground relative to the background, so that higher motif densities give rise to higher walk values in local regions of the sequence list. The RW starts over from zero every time it reaches the lower bound of − 1. This makes the RW method sensitive to local runs of enriched motifs in the sequence list.

For significance evaluation, we find the probability of a walk with at least as high a max value under the null distribution. We do this using a recursion on an analytic expression for the max value distribution of random walks (see Additional file 1: Methods for details). Alternatively, we can use a geometric-like distribution (Gumbel distribution) as an approximation for the max value distribution [24].

### Modified sum of ranks statistics

The modified sum of ranks (MSR) method is based on the idea of using a rank sum test to determine a rank bias in motif containing sequences. Rather than summing ranks, MSR uses a sum of scores specific for the sequences and motif. The scores are based on the sequence specific p-values, which eliminates bias from sequence composition and length. All motif observations are associated with a score that reflect the probability of the motif being found one or more times in the sequence, as well as the rank of the sequence. The score can be considered as a rank normalized for the probability of observing motifs in the sequence. In detail, let $s_{1} ,s_{2} , \ldots ,s_{N}$ be a list of sequences ranked according to an experimental setting, and let $n_{i}$ denote the number of observed motifs in $s_{i}.$ Under the null model, we assume $n_{i}$ ~ $po\left( {\lambda_{i} } \right)$ with $\lambda_{i} = - ln\left( {1 - p_{i} } \right)$, where $p_{i}$ is the probability of observing at least one motif in the sequence. This follows from the probability mass function of the Poisson distribution,

$$Pr\left( {X = k} \right) = \frac{{\lambda^{k} }}{k!}e^{ - \lambda } ;$$

since $p_{i} = 1 - Pr\left( {X = 0} \right) = 1 - e^{{ - \lambda_{i} }}$ we have $\lambda_{i} = - ln\left( {1 - p_{i} } \right)$.

If we think of motif occurrences as a Poisson process, where our “time axis” is composed of consecutive intervals of length $\lambda_{i}$ ordered according to the experimental rank, motif occurrences are now, under the null hypothesis, uniformly distributed across the whole interval $\left[ {0,\lambda .} \right]$ where $\lambda . = \mathop \sum \nolimits_{i = 1}^{N} \lambda_{i}$.

We now calculate a score $r_{m}$, corresponding to the mid point of the interval (sequence) in which a motif was observed.

$$r_{m} = \frac{{\mathop \sum \nolimits_{i = 1}^{m - 1} \lambda_{i} + \mathop \sum \nolimits_{i = 1}^{m} \lambda_{i} }}{2}.$$

We associate the score with motif occurrences in the sequence list. Under the null hypothesis, the probability of observing a motif in a sequence is proportional to the interval length, and thus the expectation is that motif scores are uniformly distributed across the whole interval $\left[ {0,\lambda .} \right]$. Under the null model, the score for motif occurrences is thus normally distributed with mean $\lambda ./2$ and variance $\lambda .^{2} /12$.

We calculate the test statistic

$$W = \frac{\sqrt n }{\lambda .}\left( {\frac{{\mathop \sum \nolimits_{i = 1}^{N} n_{i} r_{i} }}{n.} - \frac{\lambda .}{2}} \right) \sim {\mathcal{N}}\left( {0,\frac{1}{12}} \right)$$

where $n. = \mathop \sum \nolimits_{i = 1}^{N} n_{i}$. The motif correlation p-value is $p = 2\left[ {1 - \varPhi \left( {\left| W \right|} \right)} \right]$.

The MSR method is faster than the others because we need only the probability of observing one or more motifs in the sequence, which can be read from the TPM of the DFA (Fig. 2c) modified so that the end state is absorbing, and thus we do not need to construct the larger embedded model.

## Results

### Combined motifs increase power

**Fig. 4:**
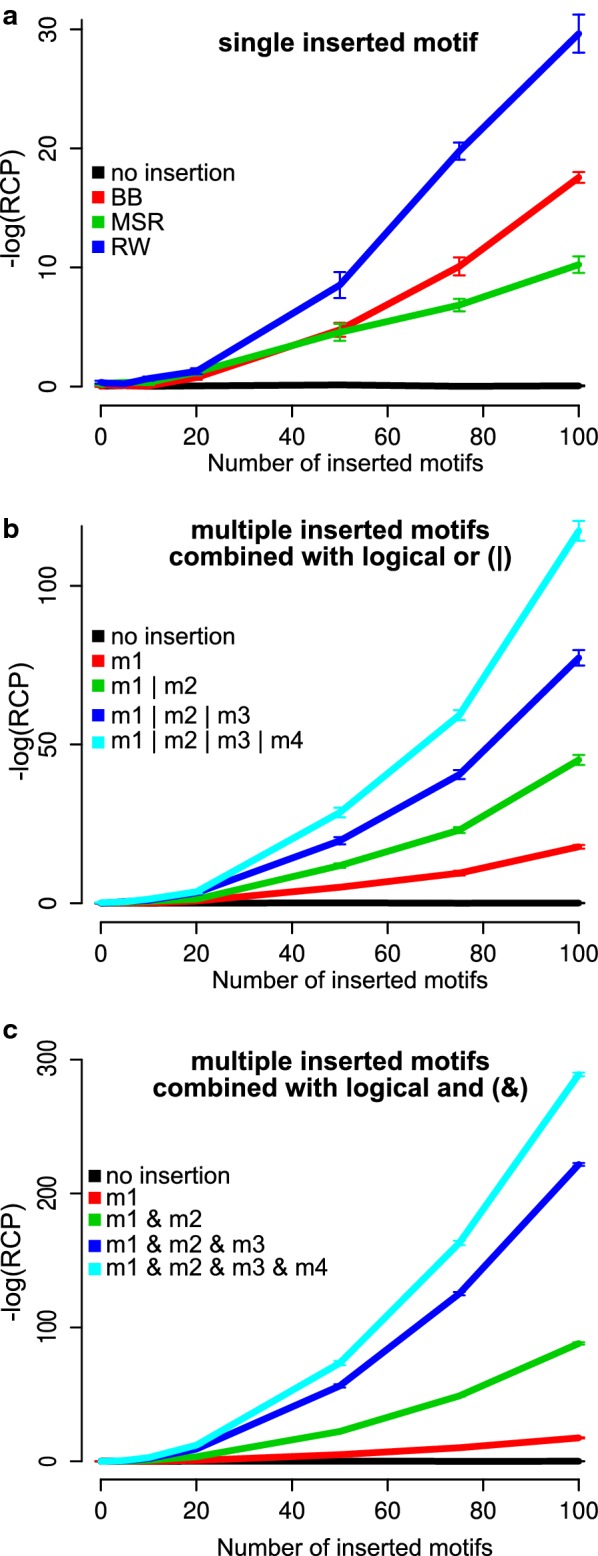
Regmex behavior in different scenarios, for 1000 sequences, each of length 1000 bases. **a** Comparison of p-value output for the different rank correlation methods used in Regmex. One 7-mer motif (ACGTGAT) is inserted as indicated in the first half of the sequences. In replicates with no insertion the BB method was used, but the other methods gave similar results. Error bars indicate standard error of 100 replicates. **b** Up to four different 7-mer motifs are inserted randomly in the first half of the sequences. p-value output from Regmex using the BB method is plotted against the number of inserted motifs. RE motifs define sets of one up to all four 7-mers as indicated, e.g., m1 | m2 = (ACGTGAT)|(GCATTGT). **c** Sets of four 7-mer motifs were inserted at fixed positions randomly among the first half of the sequences, so that motifs occur together in the same sequences. p-value output from Regmex using the BB method is plotted against the number of inserted motifs. RE motifs define sets of combinations of one up to all four 7-mers as indicated, e.g., m1 & m2 = (ACGTGATN*GCATTGT)|(GCATTGTN*ACGTGAT), where N denotes any nucleotide

Because of different characteristics of the three methods for rank correlation evaluation, they perform differently in different scenarios. We illustrate their behavior when applied to a set of 1000 random sequences with a simple 7-mer motif inserted up to 100 times in the upper half of the sequence list (Fig. 4a). In this particular scenario, the RW approach has the highest sensitivity, followed by the BB method and the MSR method. The RW method generally has a high sensitivity when the motif density is high, regardless of where in the sequence list it occurs.

This is in contrast to both the MSR and BB methods, which are more sensitive to enrichment in the beginning or end of the sequence list. The rank sum derived nature of the MSR method yields a higher sensitivity for enrichment in the ends of longer sequence lists, while the BB method is highly superior in short sequence lists with moderate enrichment (see Additional file 1: Figure S1). For extremely long sequences, such as genomes or long chromosomal segments, the Markov chain embedding underlying the BB and RW methods may become computationally demanding due to growth of the eTPM with number of motif occurrences. In such cases, the MSR method is the better choice as it depends on the simpler TPM model of a single motif occurrence, which makes enrichment calculations in long sequences less memory demanding and faster.

The use of differential scores, such as SSPs, over simple binary scores, has clear benefits. For instance, rank correlation of common and individually insignificant motifs can be better evaluated because their impact on the rank correlation is moderated by the significance of the observation. The same argument applies to rare, highly significant motifs. This, combined with RE motif definition, is useful in the case of evaluating rank correlation of combinations of motifs.

We used Regmex to evaluate rank correlation of combinations of inserted motifs in a set of random sequences. First, we inserted four different simple 7-mers up to 100 times at random positions in the upper half of the ranked sequences. We looked at the behavior of Regmex when defining motifs as REs capturing different subsets of the 7-mers including from one up to all four (i.e. REs defined to capture presence of any member of the subset). We clearly see the effect of combining multiple simple motifs in a set (Fig. 4b).

When searching for motif 1 or 2 (RE = m1|m2), we see a marked increase in detection sensitivity starting at around 20 inserted motifs. As expected, this increases with number of inserted motifs. Rank correlation increases even more dramatically for the motif subsets of three or four 7-mers. We note that the SSPs become less significant when including more 7-mers in the motif, but because the number of inserted motif observations in the enriched end of the sequence list increases (up to 400 for four 7-mers vs. 100 for a single 7-mer), the RCP becomes more significant.

We next looked at the behavior of Regmex when calculating rank correlation of multiple motifs present in the same sequences. Such calculations may be relevant when two or more different factors acting on the same sequences could explain the sequence ranking. To this end, we inserted the four 7-mers together in the same sequences. This was done up to 100 times in the upper half of the sequence list.

We used Regmex to calculate RCPs for subsets of the combined motifs, i.e. RE motifs designed to capture the presence of one up to all four 7-mers in the same sequence. The SSPs now increase in significance with the number of 7-mers in the RE subset. As expected, the detection power of the combined motifs is much higher than that of a single simple motif (Fig. 4c). These simulations show how more complex motifs, such as motif sets, can be captured by REs with great increase in power.

### U-rich motifs and miRNA seed target sites as combined motifs

As an example of a scenario where combinations of motifs are relevant, we looked for rank correlation of miRNA seed site targets in combination with a U-rich motif (URM) in a number of miRNA over-expression data sets. URMs are known to bind HuD/ELAVL4 [25] and their presence in 3′UTRs has been shown to correlate with down regulation in several miRNA over-expression experiments [21]. Based on this finding, a model was proposed where URMs augment miRNA induced destabilization of target mRNAs [21]. We used Regmex to calculate RCPs for 7-mer miRNA seed site targets and combinations of the target and the URM with sequence UUUUAAA, as identified in [21]. This was done using 11 different miRNA over-expression data sets [26, 27].

We first calculated RCPs for the miRNA seed site targets in 3′UTR sequences. For all data sets, we saw low RCPs for the miRNA seed site target corresponding to the overexpressed miRNA, demonstrating a correlation between the motif and down-regulated genes (Table 1). We next calculated RCPs for the miRNA seed site targets and URM in combination. To this end, we constructed REs of the form (UN*S)|(SN*U), where U denotes the URM, S denotes the miRNA seed site target, and N denotes any nucleotide. This RE will capture all combinations of the URM and the seed site in either order. As expected, based on the previous findings [21], we consistently saw an even lower RCP for the RE motif capturing both the seed target and the URM (Table 1). The experiment thus verifies earlier results showing URM 3′UTR presence correlating with down-regulation.

**Table 1:** Rank correlation p-values for URM (U) and seed target (S) motifs

miRNA	Seed target (S)	(SN*U)|(UN*S)	UN*S	SN*U	Refs.
miR-7	2.6e−03	1.5e−13	2.4e−11	9.6e−05	[26]
miR-9	6.6e−09	1.5e−17	2.6e−19	1.2e−05	[26]
miR-16	1.8e−178	7.3e−147	5.7e−65	9.2e−76	[27]
MiR-106b	2.5e−99	9.7e−158	4.5e−145	1.8e−58	[27]
MiR-122a	3.2e−02	4.1e−05	7.6e−04	2.7e−02	[26]
MiR-128a	6.6e−19	2.2e−48	4.7e−33	8.2e−21	[26]
MiR-132	2.4e−08	3.7e−27	3.9e−33	1.2e−07	[26]
MiR-133a	5.1e−04	9.4e−09	5.7e−06	4.3e−03	[26]
MiR-142	2.4e−05	1.4e−13	1.0e−11	6.0e−05	[26]
MiR-148b	6.3e−09	1.9e−11	3.2e−12	3.4e−04	[26]
MiR-181a	7.9e−17	3.9e−53	2.4e−46	5.1e−18	[26]

p-values for RE motifs involving URM (U) and miRNA seed site targets (S) in different combinations for miRNA over-expression data sets. All *p*-values were calculated with the Brownian bridge method. N denotes any nucleotide

We next asked whether RCPs are of similar magnitude when the URM is downstream or upstream of the seed target. Here we used Regmex with two REs: SN*U for a downstream URM and US*S for an upstream URM. We observed low RCPs for both the downstream and upstream case for all miRNAs, indicating that URM correlates with down-regulation regardless of its relative position to the seed target (Table 1). Notably, we found that RCPs were lower when the URM was found upstream of the seed target compared to downstream. This could indicate a stronger effect of the miRNA when the URM is located upstream.

The example above illustrates how Regmex can be used to test well-defined hypotheses involving combinations of motifs defined as REs. The results confirm earlier findings and further suggests that the strength of the effect of U-rich motifs on miRNA regulation is moderated by the relative position upstream or downstream of the target sites.

### Comparison and time complexity

As stated above, Regmex differs from other tools in not being a motif finding algorithm, but rather a tool for evaluating hypotheses about given motifs. A comparison with existing tools is thus restricted to scenarios where we can directly compare the output of Regmex with the output of an existing method. This is possible for the Sylamer method [1] which can return p-values for a complete set of k-mers as output, which is feasible with Regmex as well. We compared the two methods using data from a miRNA experiment in which miR-430 was injected into zebrafish [28]. The same data set was used in the original Sylamer manuscript [1]. We evaluated rank correlation p-values for all 4096 6-mer motifs. We used both the original data set rank and a randomly re-sampled gene rank to simulate a data set without any rank enriched motifs. Finally we used this re-sampled data set as the background for a spike-in run where we added an enrichment of a 10-mer motif.

**Fig. 5:**
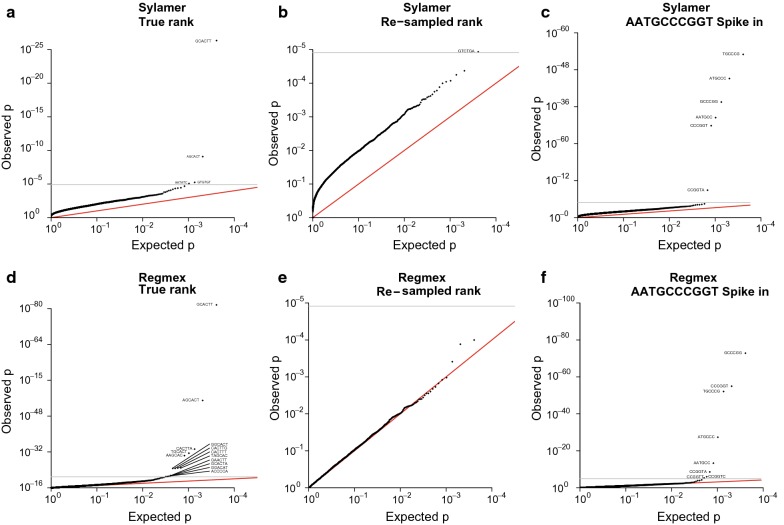
Quantile-quantile plots for observed and expected p-values of 6-mer motif runs. **a**–**c**, runs using Sylamer. **d**–**f**, runs using Regmex with the Modified Rank Sum method. The comparison employed a data set of mir-430 overexpression in zebrafish that was also used in the original presentation of Sylamer. In **a** and **d**, the un-modified data set was used. In **b** and **e**, the gene rank was randomly re-sampled. In **c** and **f**, the 10-mer AATGCCCGGT was spiked into the re-sampled sequence rank from **b** and **e**: a single motif was inserted 100 times randomly among the top 500 ranked sequences and two motifs were inserted 50 times among the first 100 sequences. Gray lines indicate Bonferroni corrected significance at the 0.05 level

For the original gene rank, both Regmex and Sylamer found that the two 6-mers with the lowest p-value were part of the seed site target of miR-430 (AGCACTT). However, the p-values reported by Regmex were orders of magnitude lower than those of Sylamer (Fig. 5a, d). Moreover, Regmex reported an additional seven 6-mers that contained five bases of the seed with a flanking base at either side as well as two 6-mers with a single mis-match and one with two mis-matches (p < 0.05, Bonferroni corrected). Both Sylamer and Regmex found two 6-mers which were not related to the seed site, and were not similar between the methods. The Sylamer method showed a tendency to report systematically inflated p-values, although they did not reach significance once corrected for multiple testing (Fig. 5a). The Regmex tail distribution had a more balanced appearance which indicate that more motifs could be enriched, yet below the significance level. When analyzing the re-sampled data set, we saw a small but systematic inflation of all p-values reported by Sylamer, and a single motif crossed the significance threshold (Fig. 5b). The Regmex method reported p-values close to the expected random uniform distribution (Fig. 5e). Finally, the evaluation on the 10-mer spike-in data set showed that both methods were capable of finding all five 6-mers included in the spike-in (Fig. 5c, f). In addition, Sylamer reports one 6-mer with five matching bases and an A overhang, whereas Regmex reports three such 6-mers with overhangs A, C and T. Both methods have zero false discoveries. Running Regmex with the Brownian Bridge setting increased sensitivity further with lower p-values and yet another significant one base overhang 6-mer, although now a false positive 6-mer occurs (Additional file 1: Figure S3).

We further compared Regmex’ performance with that of a more recent method, DRIMust [4], which can be set to output k-mers like Sylamer, although only those with p-values less than 0.01 (Table 2). Results for individual 6-mers overlapping the 10-mer spike-in motif in the re-sampled gene list indicate that Regmex has the higher significance for all fully overlapping 6-mers, although DRIMust finds two additional 5-base overlapping motifs significant.

**Table 2:** Rank correlation p-values for URM (U) and seed target (S) motifs

	Regmex Brownian bridge	Regmex modified rank sum	DRIMust	Sylamer
GCCCGG	1.23e−230*	5.91e−70	3.07e−116	1.20e−34
CCCGGT	1.77e−210*	4.55e−52	8.19e−110	5.95e−27
TGCCCG	9.90e−200*	3.35e−49	1.23e−98	4.13e−50
ATGCCC	6.63e−125*	1.73e−24	2.09e−65	2.89e−42
AATGCC	1.76e−60*	1.90e−10	7.78e−38	1.44e−29
CCGGTa	1.91−45*	1.09e−05	6.55e−31	5.00e−06
CCGGTt	2.80e−17*	0.020	2.58e−15	n.s.
CCGGTg	6.49−11*	n.s.	1.27e−09	n.s.
CCGGTc	1.16e−06	0.0044	1.06e−11*	n.s.
tAATGC	n.s.	n.s.	0.00014*	n.s.
cAATGC	n.s.	n.s.	0.0018*	n.s.

*p*-value outputs for 6-mer motifs overlapping a 10-mer spiked into a randomly re-ordered gene list for three methods, Regmex, Sylamer and DRIMust. Motif bases overlapping the 10-mer spike-in are in capital

*n.s.* Not significant

*Most significant method

The increased sensitivity of Regmex comes at the cost of computational speed. For the runs above, the time required was 2 s per 6-mer motif on one 2.67 GHz core. Sylamer runs ~ 1000 times faster on the same hardware setup. However, the built-in parallelization in Regmex makes exhaustive screens like this feasible in minutes with an 8 core machine. The reason for the difference is likely explained by the complexity of the required operations. For Regmex, the time complexity of evaluating the sequence specific probability of observing a k-mer in a sequence of length l is $O\left( {\left( {kn_{obs} } \right)^{3} log\left( l \right)} \right)$, where $n_{obs}$ is the number of k-mer observations in the sequence. This is due to the need of lifting the embedded transition probability matrix (dimension $kn_{obs}$) to the power of $l$. In typical applications, $n_{obs}$ and $k$ are rather small. Neither Sylamer nor DRIMust have probabilitiy evaluation connected to motif observation in individual sequences, and thus cost at this level is thus associated only to the search for the motif in the sequence, $O\left( l \right)$.

## Discussion

We have introduced Regmex, an R package for analyzing the distribution of motifs in ranked sequences. The method is available as an R package (https://github.com/muhligs/regmex). Regmex differs from current motif analysis methods by combining powerful RE motif definitions with accurate sequence specific significance evaluation and three different correlation score statistics. Regmex can be customized for different settings and offers customization options such as capturing of sequence di-nucleotide dependencies and motif overlaps. Alternative outputs such as sequence specific motif probabilities (SSP) and number of observed motifs (*n*_*obs*_) combined with simple data formats and support for parallelization make Regmex well suited for a range of problems. Regmex thus expands the set of tractable motif correlation problems that current methods can handle. Although Regmex is capable of traditional exhaustive k-mer screens as other methods [1, 2], it is designed for testing specific, potentially complex, motif oriented hypotheses that arise from functional genomics experiments. In particular, Regmex can accurately evaluate rank correlation significance for arbitrary combined sets of previously defined simple motifs, such as sets of binding k-mers from an unrelated transcription factor binding experiments or combinations of miRNA seed sites. It is important that such sets are from a different experiment to avoid circularity, i.e. not from e.g. a motif discovery analysis on the same data set. This is also relevant for investigations of competitive endogenous RNAs, snoRNA target sites, etc. Such motifs are not easily defined with other models such as PWMs, which also lack the position dependency structure present in a regular expression model. That said, PWM models defines full distributions over k-mers and have become the standard model for transcription factor binding sites; they would thus be relevant to include in future versions of the software.

Regmex offers three alternative ways of evaluating motif rank correlation, which differ in their null models. For the RW method, the null model is that motifs occur at random given the sequence compositions and lengths. The RW method is sensitive to stretches of low SSPs anywhere in the sequence list, and thus may find use in special cases where enrichment is expected off the ends. This could be the case if a sequence list represents consecutive functional sets of sequences, such as a gene ontologies or expression clusters. Both the MSR and the BB methods are more sensitive to motifs occurring in the ends of the list, but have subtle differences in their null models. For the MSR method, the number of observed motifs in the sequence list is fixed, and only their distribution among the sequences varies under the null. For the BB method, the null is a uniform distribution of SSPs. Although this would suggest a bias for motifs occurring more frequently than expected, the transformation of SSPs into a Brownian bridge via a running sum normalizes for this effect. Thus both of these methods should be robust to motif occurrence bias. As noted (Fig. 3 and Additional file 1: Figure S1), the MSR and BB methods have different sensitivity in different scenarios. The MSR method tends to be more sensitive than the BB method for longer sequence lists and vice versa.

The accuracy introduced by the embedded model comes at the cost of computational speed. Complex motifs can sometimes lead to large DFA models, which may cause memory use problems [29]. This is a known issue for DFAs built to recognize REs. Even REs with manageable DFAs can lead to memory use issues when using the embedded models of the BB and RW approaches, since the model grows with number of motif observations. The example presented for the G-quadroplex motif has 80 states, and if observed *n* times will give rise to an 80 × *n* eTPM model. Ideas to avoid this have been presented previously [29, 30]. In practice, however, there is often a negative correlation between motif complexity (i.e. TPM size) and sequence length, so that the eTPM matrix will tend to lie in a manageable size range. One can think of examples where such scenarios do not apply, however. Short motifs appearing often in long sequences would yield a potentially large eTPM. In such scenarios, it is advisable to either use the MSR method, which make use of the TPM rather than the larger eTPM.

Regmex is intended for applications where concrete hypotheses about motifs are evaluated on genomics data. It is possible, as illustrated in the comparison section, to perform exhaustive screens of simple motifs such as k-mers. The user should note that the output of Regmex is raw p-values, and when doing screens with multiple motifs, a multiple testing correction procedure is needed. The user should therefore employ a proper multiple testing correction, e.g. a Bonferroni correction, following motif evaluations.

Generally, motif analyses of data sets where ranking of DNA or RNA sequences may be explained by measured factors such as gene expression, holds promise to reveal novel biological insights. This is particular true if applied across large data sets. Regmex facilitates this type of analysis because rather than finding motifs, it is aimed at analyzing motifs. In contrast to other tools, this means that quantitative rank correlation outputs from Regmex can be used as a variable to correlate with other measured factors across many samples. Regmex can for instance be used to draw a full landscape of motif correlations for all k-mers across many samples. Such type of analysis may give sufficient data points to reveal novel association between motifs and correlated factors.

## Additional file

**Additional file 1.** Additional Figures and Methods.
