# Supplementary material for: Regmex: a statistical tool for exploring motifs in ranked sequence lists from genomics experiments
Source: Algorithms Mol Biol. 2018 Dec 8;13:17. doi: 10.1186/s13015-018-0135-2 (PMC6286601; doi:10.1186/s13015-018-0135-2)
Supplement: Supplementary file 1 — Additional file 1. Additional Figures and Methods. [file 13015_2018_135_MOESM1_ESM.pdf]

# Additional Material for
# Regmex: a statistical tool for exploring motifs in ranked sequence lists from genomics experiments

Morten Muhlig Nielsen [1], Paula Tataru [2], Tobias Madsen [1], Asger Hobolth [2], Jakob Skou Pedersen [1,2]

[1]Department of Molecular Medicine (MOMA), Aarhus University Hospital, Brendstrupgårdsvej 100, 8000 Aarhus C, Denmark and [2]Bioinformatics Research Centre, C.F. Møllers Allé 8, Aarhus University, 8000 Aarhus C, Denmark

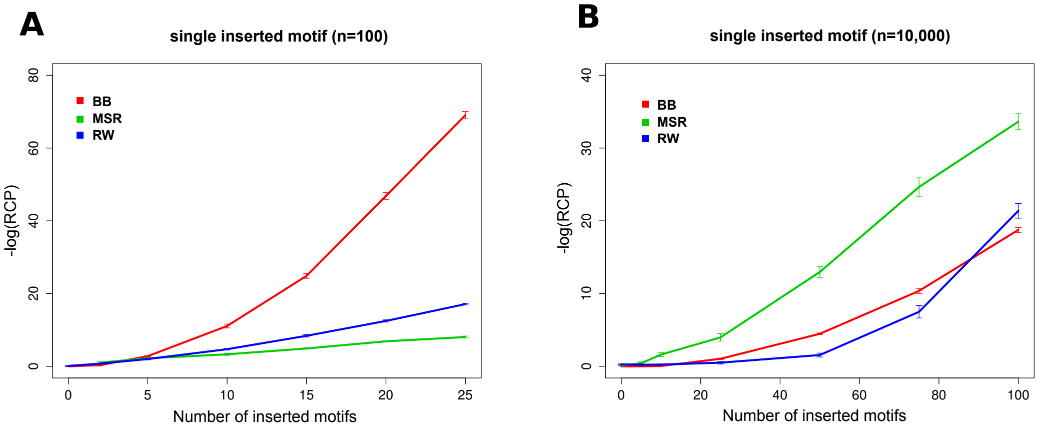


Figure S1: Regmex $p$-value output for different scenarios of sequence lengths, number of sequences and motif enrichements. **(A)** Random sequences (n=100) of length 100 nucleotides with a number of 7-mer motif (AATGCCC) inserted among the leading 30 sequences as indicated along the x-axis. **(B)** Random sequences (n=10,000) of length 100 nucleotides with a number of 7-mer motif (AATGCCC) inserted among the leading 1000 sequences as indicated along the x-axis. Bars indicate standard deviations (10 replicates)

# Methods

## Modified Rank Sum Statistic

Let $s_1 s_2, \ldots, s_N$ be a list of sequences ranked according to an experimental setting, and let $n_i$ denote the number of observed motifs in $s_i$. Under the null model, we assume $n_i \sim po(\lambda_i)$, with $\lambda_i = -\ln(1-p)$, where $p$ is the probability of observing at least one motif in the sequence. This follows from the probability mass function of the the Poisson distribution

$$Pr(X = k) = \frac{\lambda^k}{k!} e^{-\lambda};$$

since $p = 1 - Pr(X = 0) = 1 - e^{-\lambda}$ we have $\lambda_i = -\ln(1-p)$ .

If we think of motif occurrences as a Poisson process, where our "time axis" is composed of consecutive intervals of length $\lambda_i$ ordered according to the experimental rank, motif occurrences are now, under the null hypothesis, uniformly distributed on the interval $[0, \lambda.]$, where $\lambda. = \sum_{i=1}^{N} \lambda_i$.
We now calculate a score $r_m$, corresponding to the mid point of the interval (sequence) in which a motif was observed.

$$r_m = \frac{\sum_{i=1}^{m-1} \lambda_i + \sum_{i=1}^{m} \lambda_i}{2}$$

We associate the score with motif occurrences in the sequence list. Under the null hypothesis, the probability of observing a motif in a sequence is proportional to the interval length, and thus the expectation is that motif scores are uniformly distributed across the whole interval $[0, \lambda.]$. Under the null model, the score for motif occurrences is thus normally distributed with mean $\lambda./2$ and variance $\lambda.^2/12$.
We calculate the test statistic

$$W = \frac{\sqrt{n.}}{\lambda.} \left( \frac{\sum_{i=1}^{N} n_i r_i}{n.} - \frac{\lambda.}{2} \right) \sim \mathcal{N}\left(0, \frac{1}{12}\right)$$

where $n. = \sum_{i=1}^{N} n_i$. The motif correlation $p$-value is $p = 2[1 - \Phi(|W|)]$.

## Brownian Bridge Method

This method is a re-implementation of the method developed by Jacobsen et al. [1] and recently implemented in cWords [2]. Our implementation differs in the calculation of the sequence dependent motif p-values. The method calculates the max value $D$ of a running sum of mean adjusted log scores of the ranked sequence dependent $p$-values $p_i$ ($\mathrm{SSP}_i$)
$\mathrm{r}_i = r_{i-1} + ls_i - \bar{ls}$,
where $ls_i = -\ln(p_i + \alpha)$ and $\alpha$ is a score dampening factor of $10^{-5}$. $\bar{ls}$ is the mean of the log scores.

The running sum has the form of a bridge (starting and ending in 0), and the maximum value is compared to the theoretical distribution of the absolute maximum M of a Brownian bridge under the null model [3]

$$Pr(M \geq m) = 1 - 2 \sum_{k=1}^{\infty} (-1)^k e^{-2k^2 m^2 / n}$$

where $n$ is the number of sequences in the sequence list.

## Random Walk Statistics

The random walk (RW) method is inspired by the way RW theory is used in the BLAST algorithm to estimate significance of observed homologies between sequences [4]. In that case matches and mismatches become steps in a walk. Here, it is the list of sequence specific $p$-values (SSPs) for a motif that are transformed into steps in a walk. The maximum value of the walk is compared to the probability distribution of maxima under the null model.

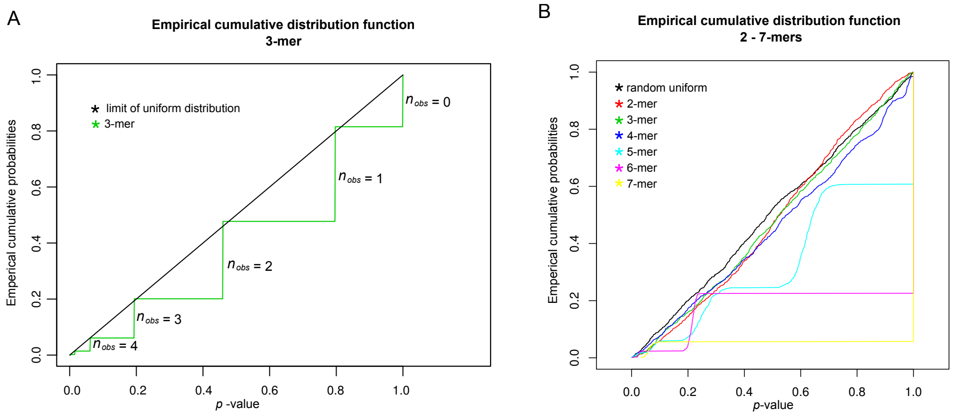


Figure S2: Empirical distributions for sequence p-values. **(A)** The plot shows the discrete behaviour of SSPs for a 3-mer motif (ATG) in 100,000 random sequences of length 100 nucleotides. All sequences were generated with equal nucleotide frequencies of 0.25. SSPs corresponding to different $n_{obs}$ are indicated. The distribution function for the SSPs define a step function that becomes nearly equal to the uniform distribution at the SSP values. **(B)** The plot shows the discrete behaviour of SSPs for random 2-7-mer motifs. The distributions were made for 1000 random sequences of 1000 bases. The sequences, unlike in (A), were generated by drawing with equal nucleotide probability, and thus do not have equal nucleotide frequencies. This is reflected in the smeared SSPs for a given motif. For longer motifs SSPs are more discrete, e.g. $p(n_{obs} \geq 0) = 1$ and $p(n_{obs} \geq 1) \sim 0.2$ for a 6-mer. Motifs used for SSP evaluation were drawn randomly from all motifs with corresponding length. A random uniform distribution [0;1] is shown for comparison.

Under the null model, sequence specific $p$-values (SSPs) are assumed to be uniformly distributed between 0 and 1. This situation is expected for (indefinitely) long random sequences. This condition, however, does not hold in general. For shorter sequences and longer motifs, the SSPs tend to discretize to small intervals specific for the number of observed motifs (see Figure S2). In particular, the $p$-value for observing "0 or more" motif occurrences is always 1. However, for random sequences, the cumulative distribution of discrete SSPs approximates that of the uniform distribution in the SSP values [5] (see Figure S2). Because the discrete SSPs depend highly on both the sequence and the motif, we normalize for the discrete effect. This is done by drawing $p$-values from the interval between the probability of observing $n_{obs}$ or more and $n_{obs}+1$ or more motifs. E.g. for a 6-mer not observed in a 1000 bases random sequence, we draw between $p(n_{obs} \geq 0) = 1$ and $p(n_{obs} \geq 1) = 0.2$. In this way the modified SSPs for random motifs in random sequences will follow the uniform distribution as required under the null model.

The modified $p$-values are now transformed into scores in a walk according to a scoring scheme based on the expected motif density when the motif is enriched (foreground) relative to the background. The scoring scheme ensures a negative drift under the null model by taking steps defined by the likelihood ratio:

$$SS(x) = \begin{cases} \lfloor s_+ \rfloor & : x \in ]0; 0.05[ \\ -1 & : x \in [0.05; 1[ \end{cases}$$

Here,

$$s_+ = \frac{-\ln(\frac{fg}{bg})}{\ln(\frac{1-fg}{1-bg})},$$

$fg$ and $bg$ are expected foreground (default = 0.2) and background (default = 0.05) motif densities in the sequence list. These default values can be changed by the user.

We are interested in the distribution of maximum values that a random walk visits before absorption, under the null model. The random walk is a particular case of a discrete-time Markov chain. We calculate the distribution of interest by building a Markov model with states $-1, 0, 1, \ldots, M$, where $M$ is the observed maximum height of the walk, and assign transition probabilities according to the scoring scheme. From the resulting probability distribution of maxima under

the null model, we derive the $p$-value for the observed maximum height. Alternatively, we can calculate the $p$-value by using a geometric-like (Gumbel) distribution approximation for the random walk maxima as given in [6].

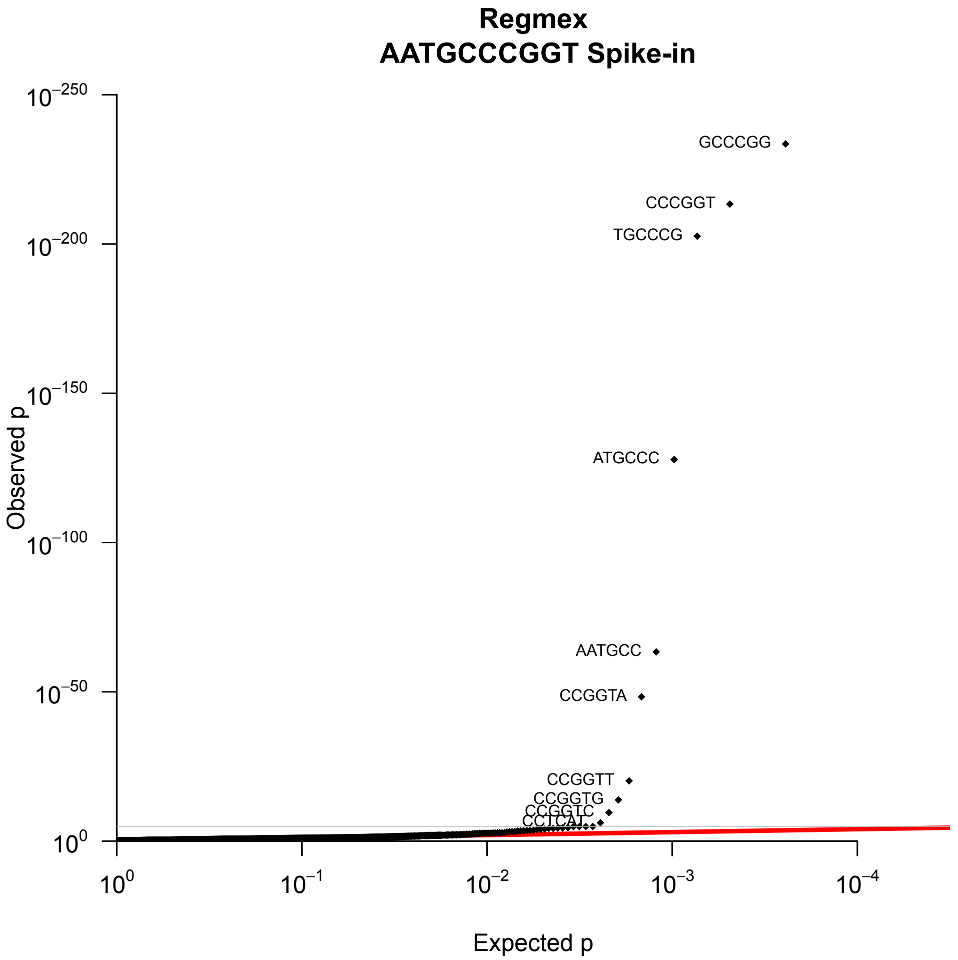


Figure S3: Quantile-quantile plots for observed and expected p-values of a 6-mer motif run using Regmex in the Brownian Bridge setting. The comparison employed a data set of mir-430 overexpression in zebrafish that was also used in the original presentation of Sylamer. The gene rank was randomly re-sampled and the 10-mer AATGCCCGGT was spiked into the re-sampled sequence rank: A single motif was inserted 100 times randomly among the top 500 ranked sequences and two motifs were inserted 50 times among the first 100 sequences.

# References

[1] Jacobsen, A., Wen, J., Marks, D. S., and Krogh, A. (2010) Signatures of RNA binding proteins globally coupled to effective microRNA target sites.. *Genome research,* **20**(8), 1010–9.

[2] Rasmussen, S. H., Jacobsen, A., and Krogh, A. (2013) cWords - systematic microRNA regulatory motif discovery from mRNA expression data.. *Silence,* **4**(1), 2.

[3] Billingsley, P. (2008) Convergence of probability measures, John Wiley  Sons, Inc., 2 edition.

[4] Altschul, S. F., Gish, W., Miller, W., Myers, E., and Lipman, D. J. (1990) Basic Local Alignment Search Tool. *Journal of molecular biology,* **215**, 403–410.

[5] Murdoch, D. J., Tsai, Y.-L., and Adcock, J. (2008) P -Values are Random Variables. *The American Statistician,* **62**(3), 242–245.

[6] Ewens, W. and Grant, G. (2005) Statistical methods in bioinformatics, Springer Netherlands, 2 edition.
